# Zero-Fluoroscopy Catheter Ablation of Right Appendage Focal Atrial Tachycardia in a Pregnant Woman

**DOI:** 10.3390/clinpract14030075

**Published:** 2024-05-21

**Authors:** Federica Troisi, Noemi Valenti, Federico Quadrini, Nicola Vitulano, Antonio Di Monaco, Imma Romanazzi, Rosa Caruso, Rocco Orfino, Massimo Grimaldi

**Affiliations:** 1 Department of Cardiology, General Regional Hospital “F. Miulli”, Acquaviva delle Fonti, 70021 Bari, Italy; 2 Department of Cardiology, A.U.O. Policlinico “Gaspare Rodolico”, 95100 Catania, Italy; 3 Department of Clinical and Experimental Medicine, University of Foggia, 71100 Foggia, Italy

**Keywords:** zero-fluoroscopy ablation, arrhythmias in pregnancy, right appendage

## Abstract

Background: Arrhythmias in pregnancy are complex to manage due to the teratogenic effects of many antiarrhythmic drugs and the common use of ionizing radiation during catheter ablation procedures. Furthermore, pregnant women are extremely vulnerable and difficult to treat because of the progressive physical and hormonal changes that occur during the nine months of pregnancy. Case Presentation: In this case report, we describe a complex clinical case of a 34-year-old pregnant woman who was affected by an incessant right atrial tachycardia, with signs and symptoms of initial hemodynamic instability. This tachycardia was refractory to antiarrhythmic drugs, so a zero-fluoroscopy ablation was performed. The first procedure was complicated by cardiac tamponade, quickly resolved without further complications for the mother or the fetus. In the following days, a deep venous thrombosis occurred at the femoral venous access. After a few days, the patient underwent a second procedure that was successful and resulted in the restoration of a sinus rhythm. Conclusions: The management of this clinical case was complex both from a procedural and a clinical (cardiological and gynecological) point of view. Finally, the integration of the various skills led to an excellent result.

## 1. Introduction

Arrhythmias in pregnancy represent a complex problem to manage, both because they involve the risk of maternal and fetal complications and because their pharmacological and interventional management must consider the possible teratogenic effects and adverse events that the fetus may encounter. Drug therapy with antiarrhythmic drugs can be partially effective in some types of arrhythmias, and, for this reason, recent guidelines indicate the possibility of performing catheter ablation procedures in selected cases and in expert centers [1].

## 2. Case Description

**Figure 1:**
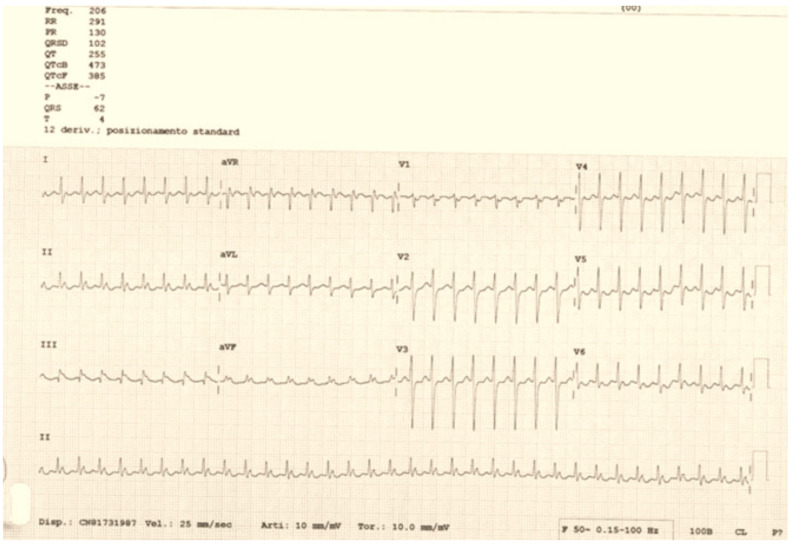
Admission ECG with right atrial tachycardia at 200 bpm.

A 34-year-old woman, 20 weeks pregnant, hypothyroid, and on hormone replacement treatment, was admitted to our hospital. The patient had been previously subjected to three different ablation procedures for right atrial tachycardia and a slow nodal pathway in 1999, 2008, and 2014. Furthermore, she reported a history of paroxysmal atrial fibrillation. The woman, during pregnancy, presented a recurrence of palpitations, with an electrocardiographic finding of right atrial tachycardia at a ventricular frequency of 200 bpm, unresponsive to vagal maneuvers or to the administration of antiarrhythmic therapy (adenosine, flecainide, metoprolol, and digoxin). When the patient arrived at our department, she presented with symptoms (palpitations and asthenia) and persistent arrhythmia with a high ventricular response (Figure 1), leading to hemodynamic instability.

**Figure 2:**
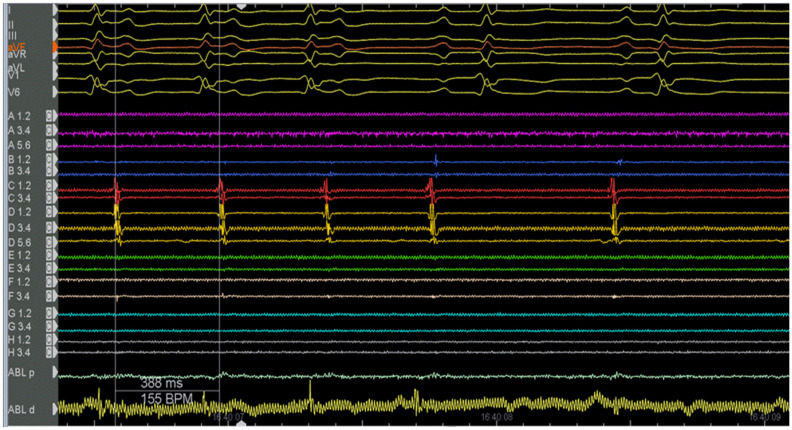
Intracavitary electrocardiographic trace of tachycardia using an Octaray mapping catheter and an ablation catheter during the first procedure.

**Figure 3:**
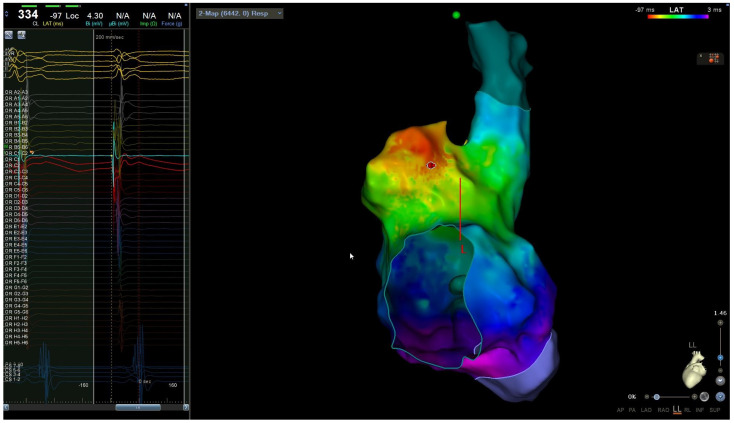
Left lateral view showing local unipolar and bipolar electrograms at the first radiofrequency application site of the earliest site.

**Figure 4:**
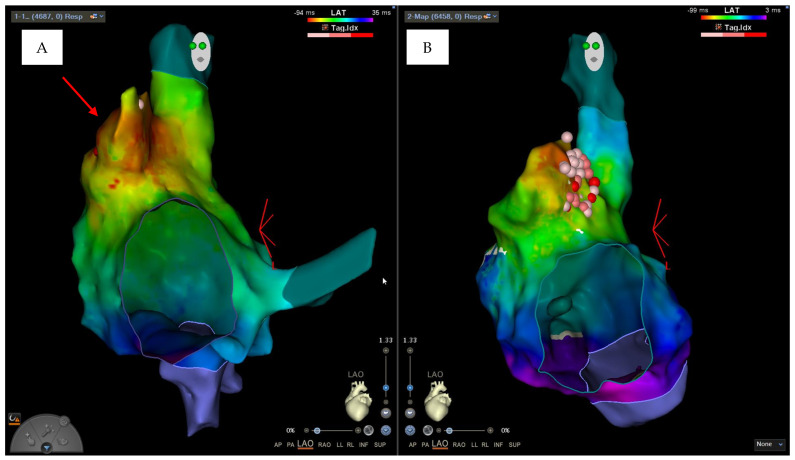
(**A**) Left anterior oblique view before ablation (arrow indicates right appendage). (**B**) Left anterior oblique view after first ablation (red and pink dots are the points of ablation).

This atrial tachycardia was refractory to antiarrhythmic therapies; as such, it was decided to subject the woman to radiofrequency catheter ablation. The procedure was performed without the use of ionizing radiation (“zero-fluoroscopy” approach). Two right femoral venous accesses were obtained with an ultrasound-guided technique, and the electrophysiological study was based on the analysis of the intracavity traces (Figure 2) integrated with an electroanatomical mapping system (CARTO 3). Two catheters were used: a mapping catheter and an ablation catheter. The Octaray mapping catheter (Biosense Webster, Inc., Irvine, CA, USA) highlighted a focal right atrial tachycardia with evidence of maximum precociousness in the right appendage; as such, radiofrequency application was started on the appendage ostium (Figure 3). A Q-DOT ablation catheter (Biosense Webster, Inc., CA, USA) was used with a maximum power of 40 watts, with an automatic control of the power based on the temperature; the contact force remained between 5 and 10 g (Figure 4A,B).

Unfortunately, during the radiofrequency applications, there was a sudden onset of marked hypotension with echocardiogram evidence of pericardial effusion and cardiac tamponade. After prompt antagonization of the 5000 IU of sodium heparin administered during the procedure with protamine sulphate, pericardiocentesis was performed with drainage of 450 mL of venous blood. The ablation procedure was halted halfway. After hemodynamic stabilization and a gynecological consultation, which documented that the fetus remained in good condition, the patient was taken to intensive care for continuous monitoring. In the following days, she underwent serial echocardiograms with evidence of stability of the pericardial effusion; a right femoral echo color Doppler documented a non-occlusive thrombosis of the common femoral vein (at the catheter insertion site). For this reason, after gynecological consent, therapy with low-molecular-weight heparin was started; serial gynecological consultations were performed, determining good fetal conditions. After 5 days, in consideration of the persistence of atrial tachycardia with high ventricular response (160 bpm), hemodynamic instability (arterial pressure of <90/65 mmHg), symptoms, and refractoriness to antiarrhythmic therapy (metoprolol and digoxin), the patient underwent another catheter ablation procedure.

The new ablation procedure was performed with an ultrasound-guided technique through two left venous accesses because of the non-occlusive thrombosis on the right femoral vein. The zero-fluoroscopy approach was based on the intracavity traces and the electroanatomical mapping system, with the support of intracardiac echocardiography, which documented the absence of thrombotic formations in the right atrium and highlighted the presence of a right appendage with very thick trabeculae.

**Figure 5:**
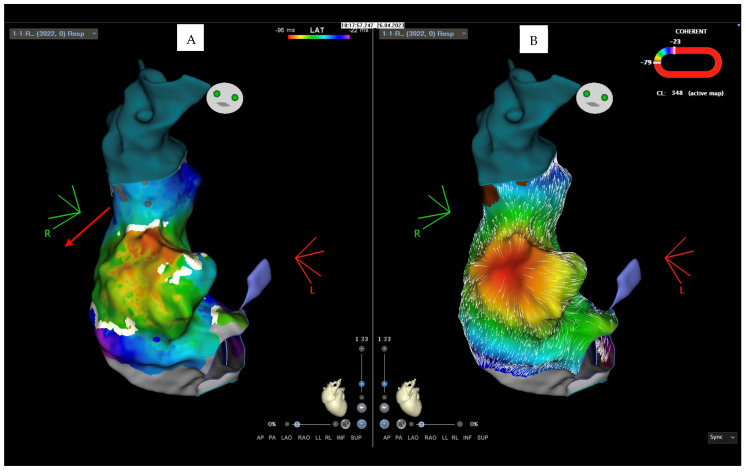
(**A**) Right lateral view of the substrate electroanatomic map of the right atrium (arrow indicates right appendage). (**B**) Right lateral view of the activation electroanatomic map of the right atrium (Coherent Algorithm).

**Figure 6:**
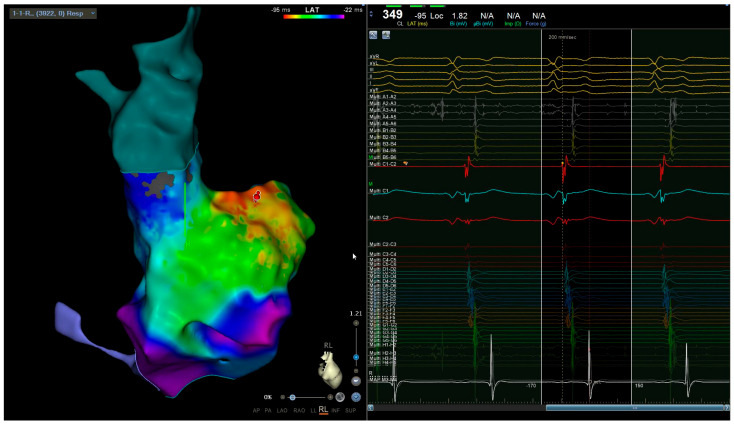
Left lateral view showing local unipolar and bipolar electrograms for the second ablation early site.

During the intracavitary electrophysiological study, the “Coherent Algorithm” of the CARTO system confirmed an atrial activation originating from the right appendage (Figure 5A,B). Radiofrequency applications in this area under intracardiac echocardiography guide led to the interruption of the circuit and the restoration of sinus rhythm, without further complications (Figure 6).

**Figure 7:**
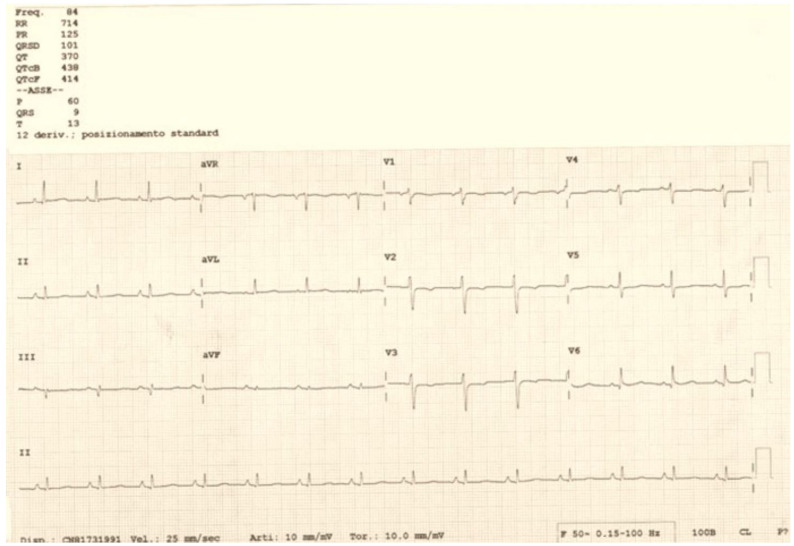
Discharge electrocardiogram of the patient.

In the following days, sinus rhythm persisted, along with the recovery of hemodynamic stability and the disappearance of symptoms, always with good fetal conditions at gynecological checkups. After one week of clinical and instrumental intensive monitoring, the woman was discharged to her home in sinus rhythm (Figure 7) and with a pharmacological prescription of low-molecular-weight heparin therapy. An ultrasound control 20 days after discharge showed the disappearance of both the pericardial effusion and femoral thrombosis, with persistence of the sinus rhythm. At 37 weeks of pregnancy, the patient gave birth to a healthy baby weighing 3.75 Kg.

## 3. Discussion

An increase in the mortality rate among pregnant women has been reported in recent years, and although the real contribution of arrhythmias to the increase in this trend has not been evaluated (as arrhythmias are often considered in the context of cardiovascular diseases in general), the rate of arrhythmias in pregnancy has also increased during the same period [2]. Furthermore, it has been demonstrated that hospitalizations for arrhythmias in pregnancy increase the rate of maternal complications (including the possible development of tachycardiomyopathy) and fetal complications (prematurity, intrauterine growth retardation, respiratory distress, and congenital heart disease) [3].

The risk of developing arrhythmias in pregnancy is related to several factors: the increasing age of pregnant women, African ethnicity, lower socio-economic status, history of cardiovascular disease or previous arrhythmias, congenital heart disease, and the presence of cardiovascular risk factors [2,4]. Pregnancy represents a vulnerable period for the development of arrhythmias for physiological reasons; the expansion of the intravascular volume causes stretching of the heart chambers, and the fluctuation in hormone levels with an increase in estrogen favors the increase in alfa-adrenergic receptors, resulting in a high sensitivity to catecholamines, increased heart rate, and increased contractility. Furthermore, oxytocic drugs, tocolytics, and peripartum anesthetics can be triggers for arrhythmias [2,3,4]. Arrhythmia’s therapy in this clinical setting is more complex than usual, as one must consider the risk of fetal adverse events and possible teratogenicity; hence, multidisciplinary management with frequent fetal monitoring is required. Many cornerstone drugs for the treatment of arrhythmias cannot be used (such as unselective beta-blockers, like atenolol, associated with higher rates of fetal growth retardation, or diltiazem, which is definitely teratogenic in animals) or must be carefully monitored in this clinical context. Adenosine, a beta-1-selective blocker, ibutilide, or flecainide seem usable, based on different levels of evidence, in the acute management of supraventricular arrhythmias, as long as they are accompanied by a medium/high surveillance level [1]. Electrical cardioversion should be considered in the case of hemodynamic instability or failure of pharmacological heart rate control. Anticoagulation can be performed with heparin in the first and third trimesters and with vitamin K antagonists in the second trimester, whereas new oral anticoagulants are contraindicated [2]. Catheter ablation should be avoided in pregnancy due to the risk of complications related to the use of fluoroscopy. It is known that the potential risks of ionizing radiation exposure to the fetus depend on the stage of pregnancy; the risks are highest during the first trimester, when the organogenesis of the central nervous system occurs. In particular, in this period, radiation exposure can lead to growth retardation, neurological diseases, and mental disorders of the fetus. However, the assumption that medical radiation doses must be kept “as low as is reasonable”, which is valid for all patients, is even more important at any stage of pregnancy. “Zero-fluoroscopic” or minimal fluoroscopic ablation (fetal radiation exposure of <1 mGy, with abdominal shielding), with the help of three-dimensional electroanatomical mapping systems and intracardiac echocardiography, should be considered in case of arrhythmias that are poorly tolerated and refractory to pharmacological therapy (Class IIA-C according to ESC guidelines) [1]. Although zero-fluoroscopy ablations are increasing, this procedure in pregnant women continues to be very challenging due to the risks, including the unknown risks that the mother and fetus may encounter during ablation. It is expected that a non-fluoroscopic technique is more likely to be considered and employed in pregnant patients at centers where there is a consistent commitment to reducing fluoroscopy for other catheter ablation procedures [5]. This is the case in our department, an electrophysiological center with a high volume of procedures and strongly oriented to a fluoro-less approach, thus limiting the use of X-rays only to ablations in which they are still truly indispensable [6].

The clinical case described was complex to manage not only due to the pregnancy itself but also due to the localization of the arrhythmic focus at the level of the right appendage. Focal atrial tachycardia originating from the right appendage represents an uncommon site of origin for focal atrial arrhythmias and causes serious problems for the execution of catheter ablation due to the characteristics of the appendix wall, which is extremely thin and vulnerable, with great variability in terms of inter-individual morphology [7,8]. The comb muscles in this site have differing thicknesses; moreover, the thin myocardial tissue between the pectinate muscles can lead to perforation and consequent cardiac tamponade during radiofrequency application. In our case, this was also favored by increased filling pressures and volume overload typical of pregnancy [9,10]. During an intracavitary electrophysiological study performed with the electroanatomical mapping system, the “Coherent software”, due to an algorithm that identifies critical conduction isthmuses of atrial tachycardias, allowed us to confirm what the electrophysiological mapping with the catheters had already hypothesized: the focal origin of atrial tachycardia from the right appendage. The QDOT Micro™ catheter (Biosense Webster, Inc., CA, USA) used during the procedures is a new radiofrequency ablation catheter that has an irrigation flow control based on temperature sensing through the six thermocouples, located within the outer metal shell. These structural characteristics of the catheter guarantee greater safety in delivery because the control of the delivered power is automatic based on the measured temperature [11]. Despite that, during the first procedure, it was probable that the thin wall and the contraction of the thick muscle trabeculae facilitated the occurrence of cardiac tamponade due to the high rate of tachycardia, which contributed to the instability of the catheter together with the position of the tip of the catheter itself between the pectinate muscles. The use of intracardiac echocardiography during the second ablation allowed for continuous checking of the catheter’s movement relative to the muscular wall of the appendage during radiofrequency delivery. Finally, the use of the “zero-fluoroscopy” technique and the help of new technologies allowed us to resolve the case without further complications for mother and fetus. The common femoral vein thrombosis that occurred after the first procedure is a possible complication expected after catheter ablation procedures using a venous approach; however, it is certain that the physiological hypercoagulable state that occurs during pregnancy facilitated this event [12]. Such a complex clinical case, burdened by two complications (intra-operative cardiac tamponade and post-operative femoral venous thrombosis), also required intensive multidisciplinary management, based on an accurate integration of both cardiological and gynecological critical issues.

## 4. Conclusions

The use of the “zero-fluoroscopic” ablation technique based on three-dimensional mapping systems and intracardiac ultrasound is increasingly expanding in the electrophysiological field. In a particular situation, such as pregnancy, these systems allow for the treatment of arrhythmias that are poorly tolerated and that are refractory to drug therapy, thus avoiding maternal and fetal complications induced by the arrhythmia while also minimizing the risk to the fetus of adverse events and teratogenic damage derived from both pharmacological therapies and fluoroscopy.

## Data Availability

The raw data supporting the conclusions of this article will be made available by the authors on request.
